# Comment on “Takotsubo Cardiomyopathy: A New Perspective in Asthma”

**DOI:** 10.1155/2015/859202

**Published:** 2015-10-12

**Authors:** John E. Madias

**Affiliations:** ^1^Icahn School of Medicine at Mount Sinai, New York, NY, USA; ^2^Cardiology Division, Elmhurst Hospital Center, Elmhurst, NY 11373, USA

I read with interest the study by Marmoush et al. [1] about the 80-year-old woman with an exacerbation of her asthma who suffered Takotsubo syndrome (TTS), after using “her rescue albuterol inhaler several times.” As the authors state this is not the first case of an albuterol-triggered TTS, as shown by many similar cases of TTS published as single case reports and series of patients derived from cohorts admitted to the respiratory intensive care unit, treated with a variety of *β*_2_-adrenergic agents. I have some thoughts for the authors to consider. (1) The electrocardiogram (ECG) on admission showed complete left bundle branch block (LBBB), which they felt was new, presumably documented by comparison with a previous ECG of the patient; however, the LBBB persisted after recovery from TTS, which is at variance with the transient nature of LBBB in other patients with TTS reported in the literature heretofore, and it is conceivable that the LBBB emerged during the time intervals between the last preadmission ECG and the admission with TTS. (2) Irrespective of this, I wonder whether the authors noted any transient attenuation of amplitude of the ECG QRS complexes of the serial ECGs, from the admission ECG to the tracings recorded during hospitalization, at discharge, and finally at the 3-month outpatient follow-up, in keeping with a recently published new insight about the ECG and TTS [2]. (3) Did this patient have any history of similar excessive use of albuterol during an exacerbation of her asthma, which however did not precipitate similar symptoms or laboratory testing finding to the TTS admission? (4) In reference to the pathophysiologic mechanism(s) which led to TTS in their patient, do the authors feel that at the roots of the TTS attack was the excessive dose of albuterol, impacting directly the cardiomyocytes, or that the externally introduced catecholamine triggered an autonomic adrenergic surge, via the sympathetic nervous system and/or adrenal catecholamine secretion? We need to start delving in such questions if we have any hopes that we will eventually understand the pathophysiological underpinnings of this mysterious affliction.
